# Supplementary material for: Unconventional charge order in a co-doped high-Tc superconductor
Source: Nat Commun. 2016 Sep 8;7:12775. doi: 10.1038/ncomms12775 (PMC5025527; doi:10.1038/ncomms12775)
Supplement: Supplementary Information — Supplementary Figures 1-6, Supplementary Note 1 and Supplementary References [file ncomms12775-s1.pdf]

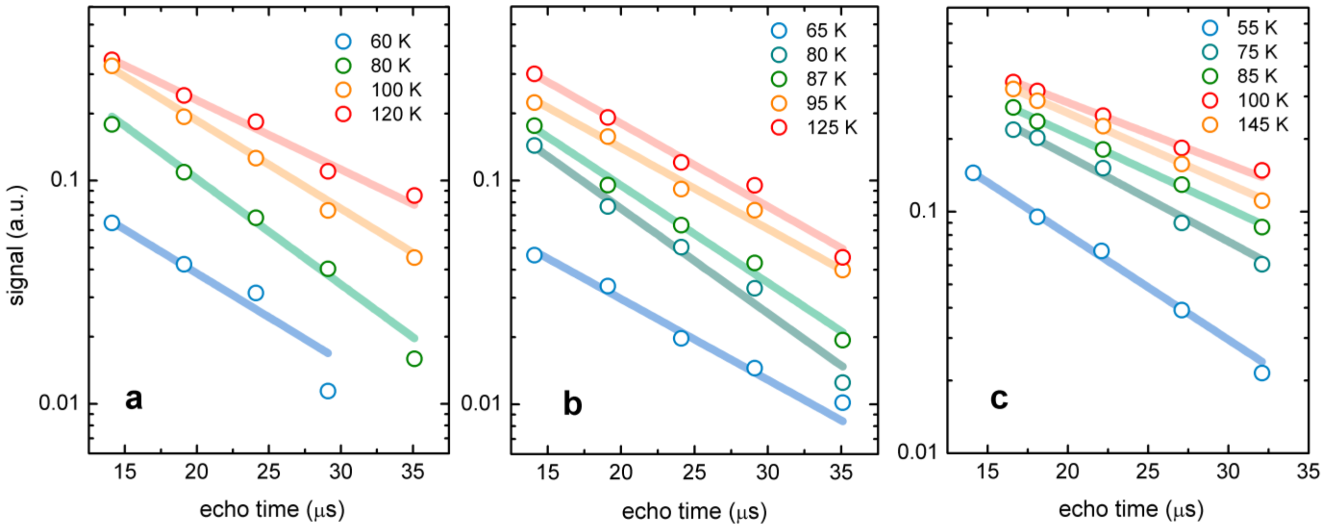

Supplementary Figure 1. Spin-spin relaxation curves for three $La_{1.8-x}Eu_{0.2}Sr_xCuO_4$ samples.

The data here are raw nuclear quadrupole resonance (NQR) data multiplied by temperature to compensate for the usual Boltzmann factor. The wipeout below the charge nematic temperature T_{EN} is clearly visible in all samples. **a** is doping $x = 0.11$, **b** is $x = 0.125$ and **c** is $x = 0.15$.

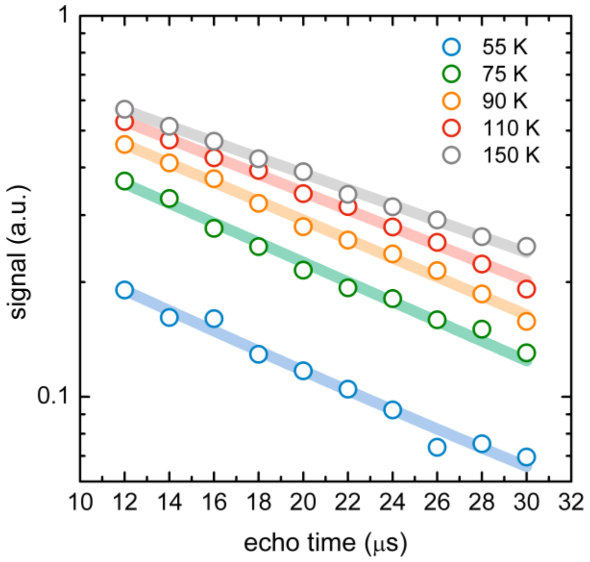

Supplementary Figure 2. Spin-spin relaxation curves for $La_{1.675}Eu_{0.2}Sr_{0.125}CuO_4$ nuclear magnetic resonance.

The relaxations are measured on the quadrupolar satellite of ^{63}Cu, at 142 MHz. Similar to nuclear quadrupole resonance (NQR), the measurements are compensated for temperature. The exponential nature of the relaxation is clearly seen, as well as wipeout similar to NQR measurements.

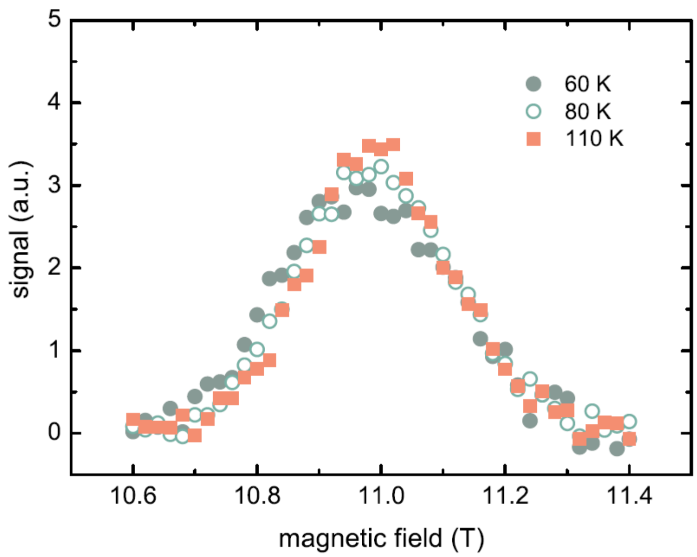

Supplementary Figure 3. Quadrupolar satellite ^{63}Cu spectra of $La_{1.675}Eu_{0.2}Sr_{0.125}CuO_4$.

Measurements were performed at 142 MHz, with a sweeped in-plane magnetic field. The spectra are normalized to their respective total areas, to highlight the similarity of spectral shapes. The similarity justifies not integrating entire spectra in obtaining the wipeout fractions.

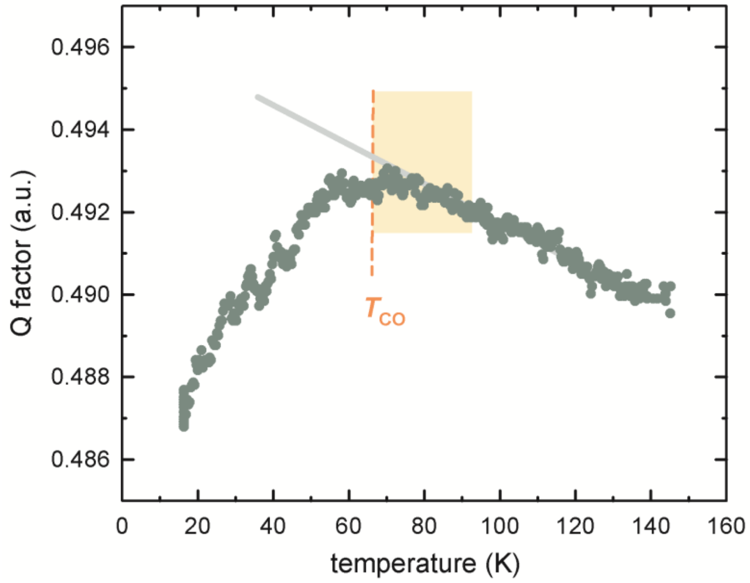

Supplementary Figure 4. In-plane AC conductivity measurement on $La_{1.8-x}Eu_{0.2}Sr_xCuO_4$.

The linear conductivity of selected $La_{1.8-x}Eu_{0.2}Sr_xCuO_4$ (LESCO) crystals was measured in the radio-frequency range, to show that the unconventional order is almost invisible in linear response. The doping concentration of the sample is $x = 0.11$, and the measuring frequency 12.4 MHz. The setup used for these measurements was very similar to the nonlinear setup described in Supplementary Ref. [1], but instead of two RF excitation/detection circuits possessed only one resonant LC circuit. Its Q-factor was significantly increased in comparison to the Q-factor of the nonlinear detection circuit of Supplementary Ref. [1] to enhance sensitivity. Thus the setup is very similar to the well-known microwave conductivity experiment [2,3] but with an LC resonator in place of a microwave cavity. The entire LC circuit was kept in liquid helium to minimize the temperature-dependent background, while the sample was in vacuum on a controlled temperature holder. The *c*-axis of the samples was aligned with the coil axis, so that circular currents were induced in the Cu-O planes, just as in the nonlinear conductivity measurement. The measured quantity is the LC circuit Q-factor, which is proportional to the square root of sample conductivity in the skin regime [2]. A strong downturn in the conductivity is observed close to T_{CO} (determined from nuclear quadrupole resonance). A slight departure from the high-temperature linear behaviour (solid grey line) is already seen several K above T_{CO}, revealing the intermediate phase in line with the results presented in the main paper. Similar features are detected in Nernst and DC resistivity measurements on LESCO with $x = 1/8$ [4,5]. Importantly, the small change in conductivity is not capable of causing a large Cu NQR signal reduction due to skin depth modification. We emphasize the sharp contrast between linear transport, which is almost featureless close to T_{EN}, and the strong divergence of the nonlinear conductivity, nicely demonstrating the unconventional nature of the ordering at T_{EN}.

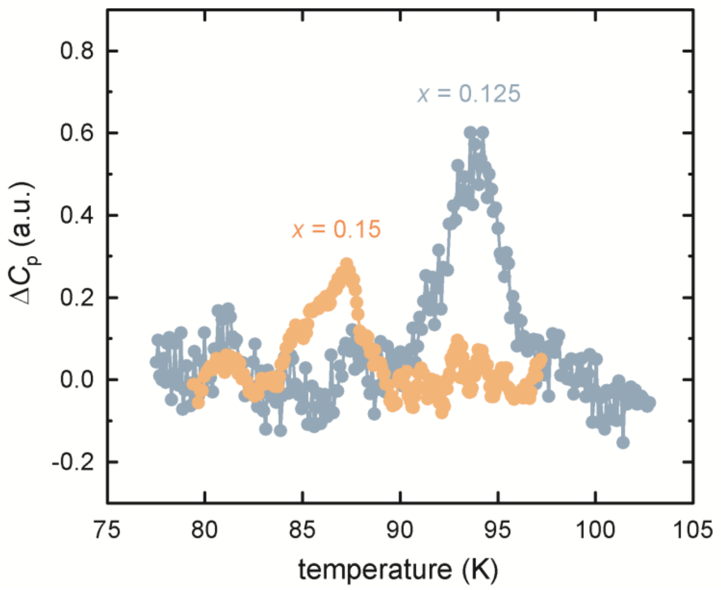

Supplementary Figure 5. Specific heat of two $La_{1.8-x}Eu_{0.2}Sr_xCuO_4$ samples.

The Sr doping is x = 0.15 (orange symbols) and x = 0.125 (blue symbols), and the data are obtained under similar experimental conditions (with two times longer filtering times for the x = 0.15 sample). The charge nematic transition temperatures correspond to the peak positions.

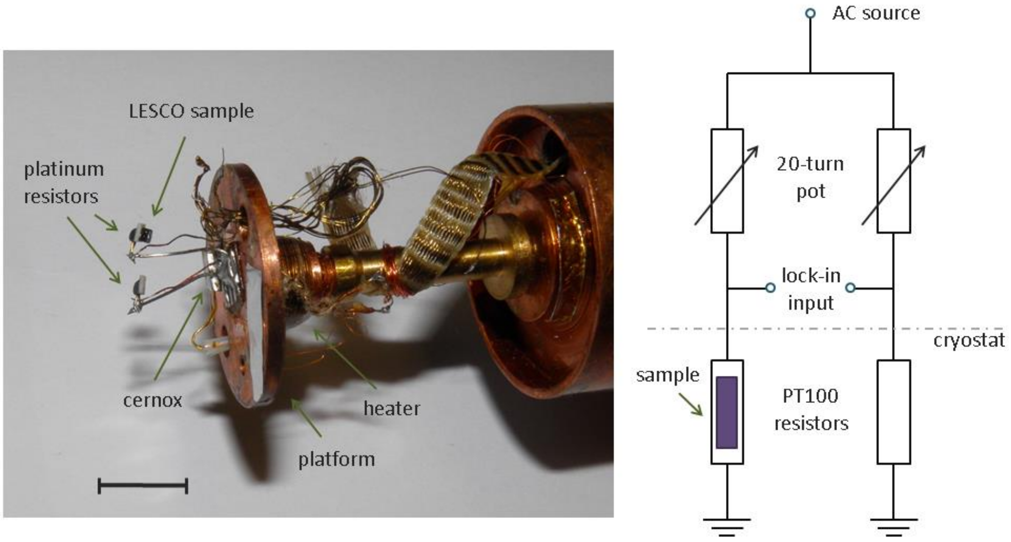

Supplementary Figure 6. The specific heat measurement setup.

a the probe head used for specific heat measurements (heatshield removed). The scale bar is 10 mm. **b** simplified electronic schematic. The two resistors are identical PT100 platinum chip resistors (standard tolerance 0.05%). Two 1kΩ 20-turn potentiometers are used to compensate for small inequalities in the two resistor circuits, and a digital lock-in amplifier was used to measure the differential voltage on the two platinum sensors.

Supplementary Note 1

Nonlinear response

The nonlinear conductivity in our experiment is measured through an induction technique - the electric fields are induced by an applied alternating magnetic field, and the nonlinear currents are also detected inductively. The sample is positioned so that both electric fields and (nonlinear) currents are in the CuO_2 planes. Designating the direction perpendicular to the planes as z, this leads to the general tensorial form of in-plane nonlinear response

$$j_{3,x} = \sum_{\alpha_1,\alpha_2,\alpha_3} \sigma_{3,x\alpha_1\alpha_2\alpha_3} E_{\alpha_1} E_{\alpha_2} E_{\alpha_3} \quad (1)$$

where α_1, α_2, α_3, = x , y, and an analogously for $j_{3,y}$. We assume perfect alignment, implying that all z-components vanish. In the experiment we detect a superposition of $j_{3,x}$ and $j_{3,y}$ (the sample is cube-shaped), and since our experiment is not phase sensitive, we cannot separate the different components in Supplementary Eq. (1). Thus in principle the x,y terms can contribute to the signal in both tetragonal and orthorhombic environments [6]. The third harmonic in our experiment is therefore not symmetry-constrained in the sense that a signal can only appear when C_4 symmetry is broken (in contrast to e.g. the well-known second harmonic in nonlinear optics, which only occurs in non-centrosymmetric media [6]). In other words, the overall signal is not proportional to the nematic order parameter (although components such as $\sigma_{3,xxxy}$ *are* symmetry-constrained). However, some components of σ_3 are a direct measure of nematic *fluctuations*, as we now discuss in more detail.

The relationship between higher-order correlations and nonlinear susceptibilities has first been established in random materials with spin-glass order [7,8], providing a generalized fluctuation-dissipation theorem (FDT) relating four-point correlations and third order response. For non-random materials, the four-point correlations entering the generalized FDT are the respective irreducible components [9]. They are proportional to quadrupolar - i.e. nematic - fluctuations, as shown in spin systems [9,10]. Our case of a charge nematic is analogous, since the symmetry of the order parameter is the same as in the spin nematic case. The fact that linear response is featureless, while the third harmonic signal diverges at T_{EN}, is direct evidence of quadrupolar/nematic ordering [9].

Qualitatively, it is easily seen that nematic charge fluctuations are related to third order response. A convenient nematic order parameter is [11,12]

$$Q_{\mathbf{k}} = \frac{S_{\mathbf{k}} - S_{\Re(\mathbf{k})}}{S_{\mathbf{k}} + S_{\Re(\mathbf{k})}} \quad (2)$$

where $S_{\mathbf{k}}$ is the static structure factor $S_{\mathbf{k}} \sim \langle \rho_{\mathbf{k}} \rho_{-\mathbf{k}} \rangle$, ρ the local charge density, and $\Re$ a rotation by $\pi/2$. Fluctuations of the nematic order parameter are then related to a susceptibility $\chi_{EN} \sim \langle S_{\mathbf{k}} S_{-\mathbf{k}} \rangle$, which clearly contains four-density correlators due to the definition of $S_{\mathbf{k}}$. We can now use the extended FDT to connect the third harmonic conductivity tensor to four-point correlators, establishing the relationship between our experimental σ_3 and nematic fluctuations. In our case of tetragonal symmetry at temperatures above the nematic transition, the components of the nonlinear conductivity tensor can be directly related to the nematic susceptibility in a simple way. We take the (linear) conductivity tensor to be diagonal, with elements $\sigma_{1,xx}$ and $\sigma_{1,yy}$; in the isotropic state $\sigma_{1,xx}$ = $\sigma_{1,yy}$ = σ_{iso}. The nematic order parameter causes an in-plane conductivity

anisotropy, and can thus be written as a scalar: $Q \sim \Delta\sigma = \sigma_{1,xx} - \sigma_{1,yy}$. Above the nematic transition, the mean value of Q is zero, but it couples to external electric fields which can induce a finite value. The appropriate field combination and corresponding nematic susceptibility is [11]

$$Q = \chi_{\mathrm{EN}}^{\sigma}(E_x - E_y)(E_x + E_y) \tag{3}$$

where we use the label $\chi_{\mathrm{EN}}^{\sigma}$ for the nematic susceptibility expressed through conductivity anisotropy. We can now calculate e.g. j_x to third order in the fields:

$$j_x \approx \left(\sigma_1^{\mathrm{iso}} + \frac{1}{2}\Delta\sigma\right)E_x = \sigma_1^{\mathrm{iso}}E_x + \frac{1}{2}\chi_{\mathrm{EN}}^{\sigma}\left(E_xE_xE_x - E_yE_yE_x\right) \tag{4}$$

A comparison to Supplementary Eq. (1) then gives relations between the components of σ_3 and $\chi_{\mathrm{EN}}^{\sigma}$ in a straightforward way. An analogous calculation can be made for j_y. Of course, in this approximate calculation we neglected all possible higher-order interaction terms, which might also contribute to j_3 and make the expressions more complicated. Yet the main conclusion, the relation between $\chi_{\mathrm{EN}}^{\sigma}$ and σ_3, remains. We note that a similar result has also been obtained for third harmonic optical response in molecular liquid crystalline liquids in the isotropic phase [13].

Supplementary References

[1] Došlić, M., Pelc, D., & Požek, M. Contactless measurement of nonlinear conductivity in the radio-frequency range. *Rev. Sci. Instrum.* **85**, 073905 (2014)

[2] Peligrad, D.-N. et al. Cavity perturbation by superconducting films in microwave magnetic and electric fields. *Phys. Rev. B* **58**, 11652-11671 (1998)

[3] Nebendahl, B., Peligrad, D.-N., Požek, M., Dulčić, A. & Mehring, M. An ac method for the precise measurement of Q-factor and resonance frequency of a microwave cavity. *Rev. Sci. Instrum.* **72**, 1876-1881 (2001)

[4] Cyr-Choiniere, O. et al. Enhancement of the Nernst effect by stripe order in a high-T_c superconductor. *Nature* **458**, 743-745 (2009)

[5] Laliberte, F. et al. Fermi-surface reconstruction by stripe order in cuprate superconductors. *Nature Commun.* **2**, 432 (2011)

[6] Boyd, R. W., *Nonlinear Optics.* (Academic Press, Burlington, 2008)

[7] Suzuki, M. Phenomenological theory of spin glasses and some rigorous results. *Prog. Theor. Phys.* **58**, 1151-1165 (1977)

[8] Bouchaud, J.-P., & Biroli, G. Nonlinear susceptibility in glassy systems: A probe for cooperative dynamical length scales. *Phys. Rev. B* **72**, 064204 (2005)

[9] Ramirez, A. P. et al. Nonlinear susceptibility as a probe of tensor spin order in URu_2Si_2. *Phys.*

Rev. Lett. **68**, 2680-2683 (1992)

[10] Morin, P., Schmitt, D. Quadrupolar effects on the third-order magnetic susceptibility. Phys. Lett. A **73**, 67-70 (1979)

[11] Kivelson, S. et al. How to detect fluctuating stripes in the high-temperature superconductors. *Rev. Mod. Phys.* **75**, 1201-1241 (2003)

[12] Chaikin, P. M. & Lubensky, T. C., *Principles of Condensed Matter Physics.* (Cambridge Univ. Press, Cambridge, 1995)

[13] Khoo, I. C. Nonlinear optics of liquid crystalline materials. *Phys. Rep.* **471**, 221-267 (2009)
